# Structural and functional hepatic factors as prognostic indicators in children with Langerhans cell histiocytosis

**DOI:** 10.3389/fonc.2026.1813004

**Published:** 2026-05-29

**Authors:** Seham Hassan, Mohamed Sedky M Sedky, Asmaa Salama, Iman Zaky, Hany Girguis, Salma Abd ELKarim, Alaa ELHaddad

**Affiliations:** 1 Pediatric Oncology Department, Children’s Cancer Hospital Egypt, Cairo, Egypt; 2 Pediatric Oncology Department, National Research Institute, Children’s Cancer Hospital Egypt, Cairo, Egypt; 3 Pathology Department, National Cancer Institute, Children’s Cancer Hospital Egypt, Cairo University, Cairo, Egypt; 4 Radiology Department, National Cancer Institute, Children’s Cancer Hospital Egypt, Cairo University, Cairo, Egypt; 5 Clinical Pathology Department, Children’s Cancer Hospital Egypt, Cairo, Egypt; 6 Clinical Research Department, Children’s Cancer Hospital Egypt, Cairo, Egypt; 7 Pediatric Oncology Department, National Cancer Institute, Children’s Cancer Hospital Egypt, Cairo University, Cairo, Egypt

**Keywords:** hepatic dysfunction, hepatomegaly, Langerhans cell histiocytosis, sclerosing cholangitis, Z-score

## Abstract

**Background and aim:**

Langerhans cell histiocytosis (LCH) is a clonal myeloid neoplasm with variable outcomes; hepatic involvement is uniformly classified as high-risk. However, liver disease includes distinct structural and biochemical phenotypes with unclear prognostic significance. This study aimed to identify prognostic determinants in pediatric hepatic LCH, define phenotype-specific risk, and assess whether Z-score–standardized liver size independently predicts disease course and treatment response.

**Methods:**

This retrospective study included 453 children with LCH treated at Children’s Cancer Hospital, Egypt, between 2007 and 2021. Patients received the LCH-III protocol before 2012 and the LCH-IV protocol thereafter. Liver involvement was evaluated using baseline biochemical tests and ultrasonography, and liver span Z-scores were calculated to standardize hepatomegaly.

**Results:**

Eighty patients had hepatic involvement: 27 presented with hepatomegaly and preserved liver function, while 53 had hepatic dysfunction. Hepatic dysfunction independently predicted initial treatment failure (OR 9.28; 95% CI, 2.21–64.3; p = 0.001) and was associated with markedly inferior 5-year overall survival (OS) (39.4% vs 92.6%) and event-free survival (EFS) (12.9% vs 70.4%) compared with those with hepatomegaly and preserved function (all p< 0.0001). In addition, hypoalbuminemia emerged as an adverse biochemical prognostic factor (HR 4.40; 95% CI, 1.52–12.7; p = 0.003). Multivariable analysis demonstrated that week 6 non-response was the only independent predictor of both OS (HR 3.17; 95% CI, 1.37–7.31; p = 0.007) and EFS (HR 9.26; 95% CI, 4.12–20.84; p< 0.001). Conversely, outcomes were not affected by the extent of hepatomegaly, suggesting that liver size alone does not confer prognostic significance in the absence of functional hepatic impairment.

**Conclusions:**

In pediatric LCH, hepatic dysfunction, rather than hepatomegaly, emerges as the true determinant of high-risk liver involvement. Lake of early response at week 6 independently predicts inferior outcomes. Children with preserved liver function demonstrate excellent outcomes irrespective of liver size. These findings suggest potential refinement of current risk definitions and support a shift toward function-based risk stratification, pending validation in larger, multicenter prospective studies.

## 1 Background

Langerhans cell histiocytosis (LCH) is the most prevalent histiocytic disorder and is defined by the clonal proliferation of pathologic dendritic cells (1). Emerging molecular and clinical evidence has reclassified LCH as a myeloid neoplasm arising from hematopoietic progenitor cells. The disease exhibits marked clinical heterogeneity, ranging from indolent, self-limited lesions to aggressive, life-threatening multisystem involvement (2). At diagnosis, LCH is stratified according to disease extent into single-system LCH (SS-LCH), involving a single organ or system, and multisystem LCH (MS-LCH), affecting two or more organ systems, with or without involvement of risk organs, including the hematopoietic system, liver, and spleen (3). Hepatic involvement is observed in approximately 10–20% of patients with LCH and is defined by structural and/or biochemical abnormalities (4, 5). In advanced disease, liver involvement is frequently complicated by sclerosing cholangitis (SC), characterized by progressive histiocytic infiltration and destruction of the biliary tree (6). Importantly, hepatic LCH is a strong adverse prognostic factor, associated with treatment refractoriness, increased risk of disease reactivation, and progressive complications such as cirrhosis and portal hypertension (7). Accordingly, reported mortality rates in this subgroup range from 30% to 50%, compared with less than 10% in those without hepatic disease (8–10). Given the disorder’s rarity, reports of hepatic LCH are limited. Its clinical features and treatment outcomes remain insufficiently explored, particularly concerning the distinction between hepatomegaly and hepatic dysfunction, two patterns of liver involvement that are frequently considered under the umbrella of high-risk organ (RO^+^) involvement in treatment protocols.

To address this knowledge gap, we conducted a 14-year retrospective cohort study at a single tertiary center to identify prognostic variables associated with outcomes in children with hepatic LCH. The principal objective was to determine whether children with hepatomegaly and preserved liver function differ prognostically from those with biochemical hepatic dysfunction. In addition, we evaluated the prognostic value of hepatomegaly using Z-score–adjusted liver span measurements to standardize liver size quantification and to assess whether the degree of hepatomegaly independently predicts disease course and treatment response.

## 2 Patients and methods

This retrospective study included patients younger than 18 years diagnosed with LCH and treated at Children’s Cancer Hospital Egypt (CCHE) between June 2007 and December 2021.

### 2.1 Study population and data collection

A total of 453 pediatric patients with LCH were identified during the study period. Patients with SS-LCH were excluded, resulting in 186 cases with MS-LCH. Of these, a consecutive cohort of 80 patients (43%) with confirmed hepatic involvement was selected for comprehensive analysis. Demographic, clinical, laboratory, imaging, treatment, and outcome data were retrospectively collected from electronic medical records. The study protocol was reviewed and approved by the Scientific and Medical Advisory Committee (SMAC) and the Institutional Review Board (IRB). Written informed consent was obtained from the legal guardians of all patients prior to initiation of chemotherapy.

### 2.2 Diagnostic and stratification criteria

Beyond clinical and radiological findings, the diagnosis of LCH was established through histopathological and immunophenotypic analysis of lesional tissue, obtained from the most accessible and representative site, demonstrating positivity for CD1a and/or CD207 (langerin) (4).

According to the Histiocyte Society classification, patients with MS-LCH were stratified into a high-risk group when risk organs (RO+) were involved and into a low-risk group when disease occurred without risk-organ involvement (RO–). RO+ status was assigned when at least one designated risk organ was involved. For liver involvement, it required the presence of one or more of the following criteria: (1) hepatomegaly, defined as a liver edge palpable more than 3 cm below the costal margin at the mid-clavicular line; (2) evidence of hepatic dysfunction, demonstrated by abnormal laboratory parameters including bilirubin >1.5× the upper limit of normal, total protein<5.5 g/dL, serum albumin<2.5 g/dL, transaminases (ALT or AST) >3× the normal range, or alkaline phosphatase (ALP) or γ-glutamyl transpeptidase (GGT) >1.5× the normal range, and/or by clinical or radiological manifestations, including intrahepatic nodular lesions, ascites, or edema not attributable to alternative causes (4, 11, 12).

Other RO+ included hematopoietic dysfunction, evidenced by bi- or tri-lineage cytopenia, splenic involvement defined as splenomegaly ≥2 cm below the costal margin, both hepatomegaly and splenomegaly were confirmed by ultrasonography (US). Pulmonary involvement was identified on computed tomography (CT) by the presence of cysts or nodules; however, following the 2012 revision of the Histiocyte Society criteria, lung involvement has been reclassified as RO– (13, 14).

### 2.3 Radiological and histopathological assessment

Abdominal US was performed in all patients as the initial imaging modality. Hepatic involvement was categorized into 2 stages: an early infiltrative stage, defined by diffuse hepatomegaly, periportal hypoechoic thickening, hypoechoic parenchymal nodules near biliary radicles, and/or absent or minimal intrahepatic bile-duct dilatation; and a late fibrotic stage, characterized by periportal fibrosis and/or segmental intrahepatic bile-duct dilatation or narrowing, with or without distortion of hepatic architecture (15). Liver biopsy specimens, when available, were evaluated for inflammatory activity and fibrosis using the Batts–Ludwig grading and staging system (16).

SC was defined as advanced cholestatic liver disease characterized by elevated direct bilirubin, increased cholestatic enzymes (GGT and/or ALP), and US findings consistent with late fibrotic hepatic involvement, in accordance with previously published criteria (12, 17).

### 2.4 Liver span Z-score:

Age-adjusted liver span Z-scores were derived from baseline US measurements using the normative reference data of Waelti et al. (18) to ensure standardized quantification of hepatomegaly and facilitate valid comparisons across age groups. Participants were categorized according to age-specific reference tables, and Z-scores were computed using the standard equation (19).


$$Z=\frac{\left(\text{X }-\text{ μ}\right)}{\text{σ}}$$

where *X* is the US-measured liver span, *μ* the age-specific mean, and σ the corresponding standard deviation. Liver-span Z-scores > +2 to< +3 SD were classified as mild to moderate hepatomegaly, while Z-scores ≥ +3 SD were considered massive hepatomegaly, in line with international and WHO anthropometric standards, which define values ≥ +3 SD as extreme (20, 21).

### 2.5 First-line treatment

Patients were managed according to Histiocyte Society MS/RO+ guidelines, following the protocol active during the study period. From 2007–2011, treatment followed the LCH-III protocol, consisting of a 6-week induction I phase with prednisone (PRED; 40 mg/m^2^/day), weekly vinblastine (VBL; 6 mg/m^2^), and intermediate-dose methotrexate (ID-MTX; 500 mg/m^2^ every other week). For patients who demonstrated an inadequate response after the first 6 weeks, induction II (an additional 6 weeks) was administered, mirroring induction I but with PRED given on days 1–3 each week. This was followed by 12 months of continuation therapy with daily 6-mercaptopurine (6-MP) and weekly oral MTX. From 2012 onward, patients were treated according to the LCH-IV International Collaborative Protocol, which retained a PRED/VBL induction backbone but omitted ID-MTX. Continuation therapy comprised combinations of vincristine, cytarabine, PRED, 6-MP, and MTX, administered over 24 months (22, 23).

#### 2.5.1 Treatment evaluation

First-line treatment response was evaluated according to International LCH Study Group criteria. Outcomes were classified as non-active disease (NAD) when all manifestations resolved; active disease better (ADB) when improvement was observed; active disease intermediate (ADI) when disease remained stable; and active disease worse (ADW) when clinical deterioration or new lesions occurred (4, 12).

Treatment failure was defined as either failure of response to initial therapy or disease reactivation. Failure of response to initial therapy was classified as ADI or ADW at the end of Induction II, or ADW at the end of Induction I. Reactivation was defined as disease progression after Induction II in patients who had previously achieved NAD or ADB (22, 23).

Early treatment response at week 6 (end of induction I) was assessed and patients were categorized as responders (NAD/ADB) or non-responders (ADI/ADW).

### 2.6 Salvage treatment

During the LCH-III era, the protocol did not specify a standardized salvage regimen for treatment failure. Consequently, patients at our center who failed to respond to initial therapy were managed with palliative or compassionate approaches, whereas those who experienced disease reactivation were treated with a repeat induction course of PRED/VBL ± ID-MTX. With the implementation of the LCH-IV protocol, both categories of patients with RO^+^ disease became eligible for protocol-defined salvage strategies, most commonly cladribine-based regimens (2-CdA BR) (23, 24).

### 2.7 Statistical analysis

Baseline characteristics, clinical outcomes, and prognosis were analyzed using appropriate descriptive and comparative statistics. Continuous variables were summarized as means (SD) or medians (range) and compared with the Kruskal–Wallis test, while categorical variables were compared using chi-square or Fisher’s exact tests. Event-free survival (EFS), overall survival (OS), and reactivation-free survival (RFS) were estimated using the Kaplan–Meier method and compared across subgroups. EFS was defined as the time from diagnosis to the first event (failure of response to initial therapy, reactivation, or death) or the date of last contact. OS was defined as the time from diagnosis to death from any cause or to censoring at the date of the last follow-up, and RFS was defined as the interval from the response post induction II to the first occurrence of reactivation, specified as disease progression overall or within risk organs. Patients without an event were censored at their last response evaluation. Cox proportional hazards regression was used to evaluate the association between clinical covariates and survival outcomes, with hazard ratios (HR) and 95% confidence intervals (CI) calculated. The proportional hazards assumption was verified using Schoenfeld residuals, with no significant violations detected. Model discrimination was assessed using Harrell’s concordance index (C-index). To minimize overfitting, the number of covariates in multivariable models was restricted according to the events-per-variable (EPV) rule (≥10 events per variable). Univariate and multivariable logistic regression analyses evaluated predictors of failure of respond to initial therapy. Model calibration was checked using the Hosmer-Lemeshow goodness-of-fit test. P-values were adjusted for multiple comparisons with the Benjamini-Hochberg false discovery rate (FDR) method. A two-sided p-value<0.05 was considered statistically significant. All analyses were performed using R version 4.5.1 (R Foundation for Statistical Computing, Vienna, Austria).

## 3 Results

### 3.1 Clinical characteristics of patients with hepatic LCH

Among 186 patients with MS-LCH, hepatic involvement was identified in 80 patients based on imaging and/or liver function tests. Within the hepatic group, 27 patients presented with hepatomegaly and preserved liver function, whereas 53 exhibited liver dysfunction; among these, 48 had concomitant hepatomegaly and liver dysfunction, and 5 had isolated liver dysfunction with normal liver size. The remaining 106 patients constituted the non-hepatic group, of whom one was classified as RO+ due to splenic involvement, while 105 were RO-. Compared with the non-hepatic group, patients in the hepatic group demonstrated a slight male predominance (55%; male-to-female ratio, 1.2:1) and were significantly younger at diagnosis, with a higher proportion diagnosed before 2 years of age (p< 0.001). Furthermore, hepatic involvement was associated with higher rates of skin, lymph node, splenic, and hematopoietic system involvement (**Table 1**).

**Table 1:** Clinical characteristics of patients with MS-LCH stratified by hepatic involvement.

Characteristics	Hepatic, n=80 N (%)	Non-hepatic, n=106 N (%)	P-Value
Age (Years)			
Mean ± SD (Min-Max)	2.02 ± 1.41 (0.20-9.15)	4.00 ± 3.23 (0.39-16.64)	<0.001
< 2	51 (64%)	28 (26%)	<0.001
≥ 2	29 (36%)	78 (74%)	
Gender			
Female	36 (45%)	43 (41%)	0.5
Male	44 (55%)	63 (59%)	
CNS risk bones			
No	45 (56%)	59 (56%)	0.9
Yes	35 (44%)	47 (44%)	
Bone			
No	22 (28%)	6 (5.7%)	<0.001
Yes	58 (73%)	100 (94%)	
Skin			
No	45 (56%)	92 (87%)	<0.001
Yes	35 (44%)	14 (13%)	
Lymph node			
No	50 (63%)	83 (78%)	0.018
Yes	30 (38%)	23 (22%)	
Pituitary			
No	77 (96%)	96 (91%)	0.13
Yes	3 (3.8%)	10 (9.4%)	
Lung			
No	67 (84%)	90 (85%)	0.8
Yes	13 (16%)	16 (15%)	
Spleen			
No	23 (29%)	105 (99%)	<0.001
Yes	57 (71%)	1 (0.9%)	
Hematopoietic system			
No	60 (75%)	106 (100%)	<0.001
Yes	20 (25%)	0 (0%)	

### 3.2 Imaging, and laboratory presentations

Imaging revealed early infiltrative hepatic stage in 60 of 80 patients (75%), most commonly diffuse hepatomegaly (n = 46), with additional infiltrative features such as periportal hypoechoic thickening (n = 8) and focal parenchymal nodules (n = 6). A late fibrotic hepatic stage was identified in 16 patients, including 4 with established cirrhosis, while 4 patients had normal liver size and no detectable US abnormalities.

Laboratory abnormalities were common. Hypoalbuminemia was the most common finding, affecting 52.5% (42/80) of patients. Hyperbilirubinemia was observed in 43.7% (35/80), including elevated direct bilirubin in 30% (24/80). Cholestatic enzyme elevation (GGT and/or ALP) occurred in 32.5% (26/80), prolonged prothrombin time in 30% (24/80), and elevated serum transaminases in 16% (13/80).

#### 3.2.1 Cholestatic liver disease with features of sclerosing cholangitis (SC)

Overall, 15 of 80 patients (18.8%) developed SC during the study period. Among the 16 patients with ultrasonographic late fibrotic changes, 13 fulfilled SC criteria based on cholestatic laboratory abnormalities. In the remaining three patients, assessment for SC was not possible owing to the absence of baseline laboratory data. Notably, two additional patients who did not meet SC criteria at baseline subsequently developed biochemical and ultrasonographic features consistent with SC during disease reactivation.

Liver biopsy was performed in 11 patients, when clinically feasible, to evaluate cholestatic liver disease and assess for features of SC. Histopathological evaluation showed findings consistent with cholangitis, including bile duct epithelial injury with ductular reaction and portal tract expansion by inflammatory cells, edema, and fibrosis. Fibrosis ranged from stage 1 to cirrhosis, with no biopsy demonstrating classic periductal onion-skin fibrosis. CD1a immunostaining was negative in all specimens except one, which showed focal positivity in a few Langerhans cells. (**Supplementary Figure 1**).

### 3.3 Treatment response

Among 80 patients with hepatic LCH, 22 (28%) received LCH-III and 58 (72%) received LCH-IV as first-line therapy. Treatment failure occurred in 53 patients (66%), including failure of response to initial therapy in 32 patients (5 LCH-III and 27 LCH-IV) and disease reactivation in 21 patients (11 LCH-III and 10 LCH-IV; RO− in 8 and RO+ in 13). Of these 53 patients, 33 (62%) received salvage therapy, including 21 treated with 2-CdA BR regimens and 12 treated with alternative protocol-based regimens. Notably, among patients treated with 2-CdA BR, 5 of 13 with failure to respond to initial therapy and 7 of 8 with disease reactivation ultimately died from progressive disease. Overall, 37 patients (46%) died during follow-up, most commonly from progressive liver disease (n = 25) or complications of SC (n = 10); one death each was attributed to sepsis and secondary malignancy. Among surviving patients, five had persistent SC, including two who subsequently underwent liver transplantation. (**Supplementary Figure 2**).

### 3.4 Factors associated with Failure of response to initial therapy

In univariate analysis, younger age at diagnosis (<2 years), liver dysfunction, and hematopoietic system involvement were associated with poor initial therapy response, whereas the induction protocol used (LCH-III vs. LCH-IV) showed no significant association. After adjustment for relevant covariates and FDR correction, liver dysfunction emerged as the sole independent factor associated with failure of response to initial therapy (OR 9.28; 95% CI, 2.21–64.3; p = 0.001) (**Table 2**).

**Table 2:** Factors associated with failure of response to initial therapy: univariate and multivariable analyses.

	Univariate			Multivariate		
Risk Variables	OR (95% CI)	P-value	q-value ^1^	OR (95% CI)	P-value	q-value ^1^
Age at diagnosis (Years)						
≥2 (Ref) <2	3.02 (1.14 - 8.79)	0.032	0.054	2.25(0.71- 7.63)	0.2	0.2
Liver Dysfunction						
No (Ref) Yes	16.3 (4.26 -108)	<0.001	0.001	9.28 (2.21- 64.3)	0.001	0.004
Associated Hematopoietic						
No (Ref) Yes	7.59 (2.52- 26.4)	<0.001	0.001	3.59 (1.09 -13.5)	0.036	0.054
Induction Therapy						
LCH -III LCH - IV	2.96 (1.02, 9.98)	0.058	0.058	Not included	–	–

1 False discovery rate (FDR) correction for multiple testing.

CI, Confidence Interval; OR, Odd Ration.

### 3.5 Survival outcomes

**Figure 1:**
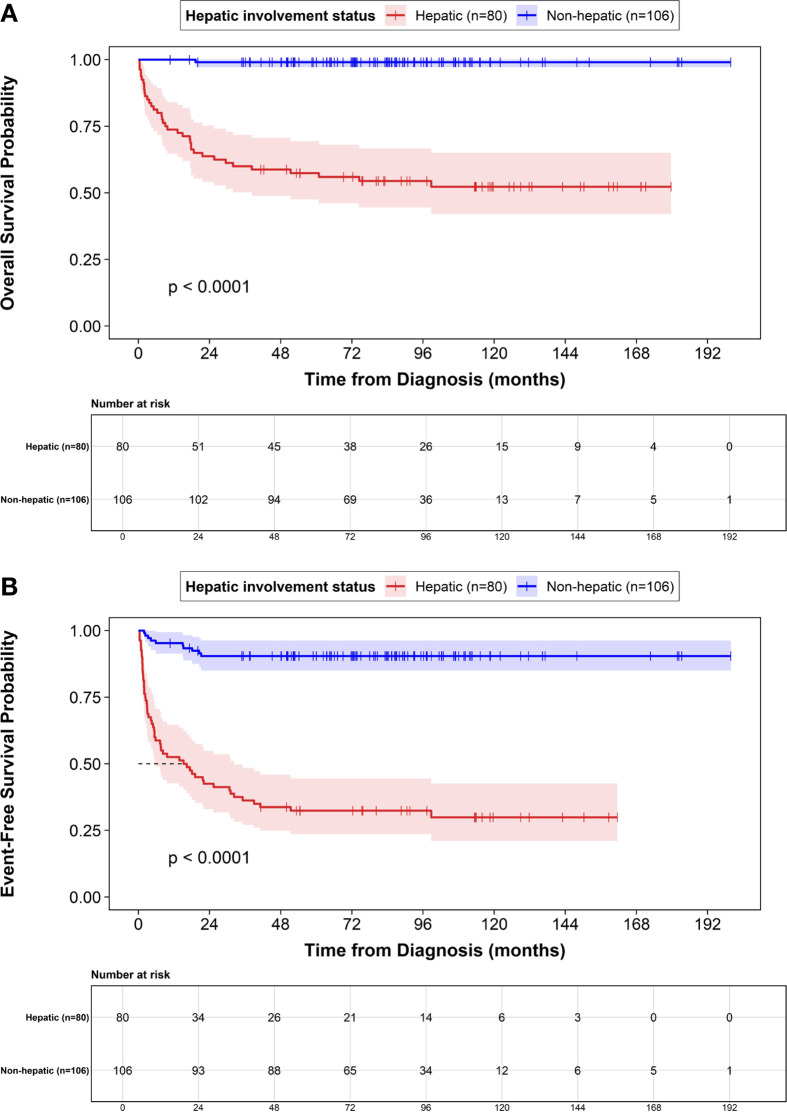
OS **(A)** and EFS **(B)** of MS-LCH patients with and without liver involvement.

The median follow-up among surviving patients with hepatic involvement was 114 months (range, 41.5–180 months). Patients with hepatic involvement experienced significantly inferior survival outcomes compared with MS-LCH patients without liver involvement. The 5-year OS rate was 57.4% ± 5.5% in the hepatic involvement group versus 99.0% ± 1.0% in those without hepatic disease, while the corresponding 5-year EFS rates were 32.4% ± 5.2% and 90.5% ± 2.9%, respectively (both P< 0.0001; **Figure 1**).

**Figure 2:**
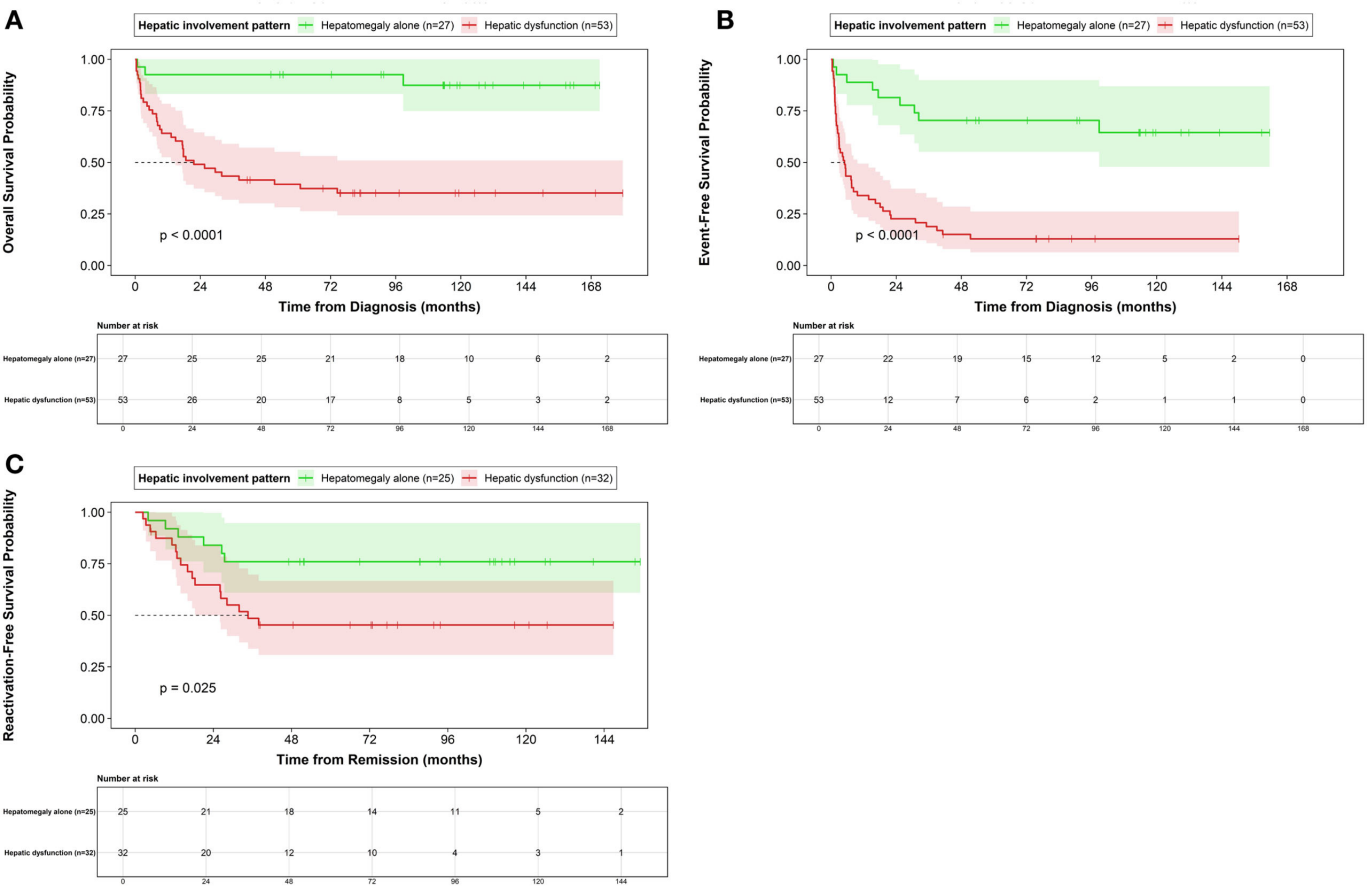
Prognostic impact of hepatic dysfunction on **(A)** OS, **(B)** EFS, and **(C)** RFS.

Notably, hepatic dysfunction was a strong adverse prognostic factor. Patients with hepatic dysfunction had markedly inferior 5-year OS (39.4% ± 6.7%), EFS (12.9% ± 4.7%), and RFS (45.3% ± 8.9%) compared with those with hepatomegaly and preserved liver function, who achieved significantly higher rates of 92.6% ± 5.0%, 70.4% ± 8.8%, and 76% ± 8.5%, respectively (**Figure 2**). Furthermore, within the hepatic dysfunction subgroup, survival outcomes (OS and EFS) did not differ significantly between patients with and without SC. (**Supplementary Figure 3****).**

#### 3.5.1 Does hepatomegaly confer additional prognostic impact beyond hepatic dysfunction?

**Figure 3:**
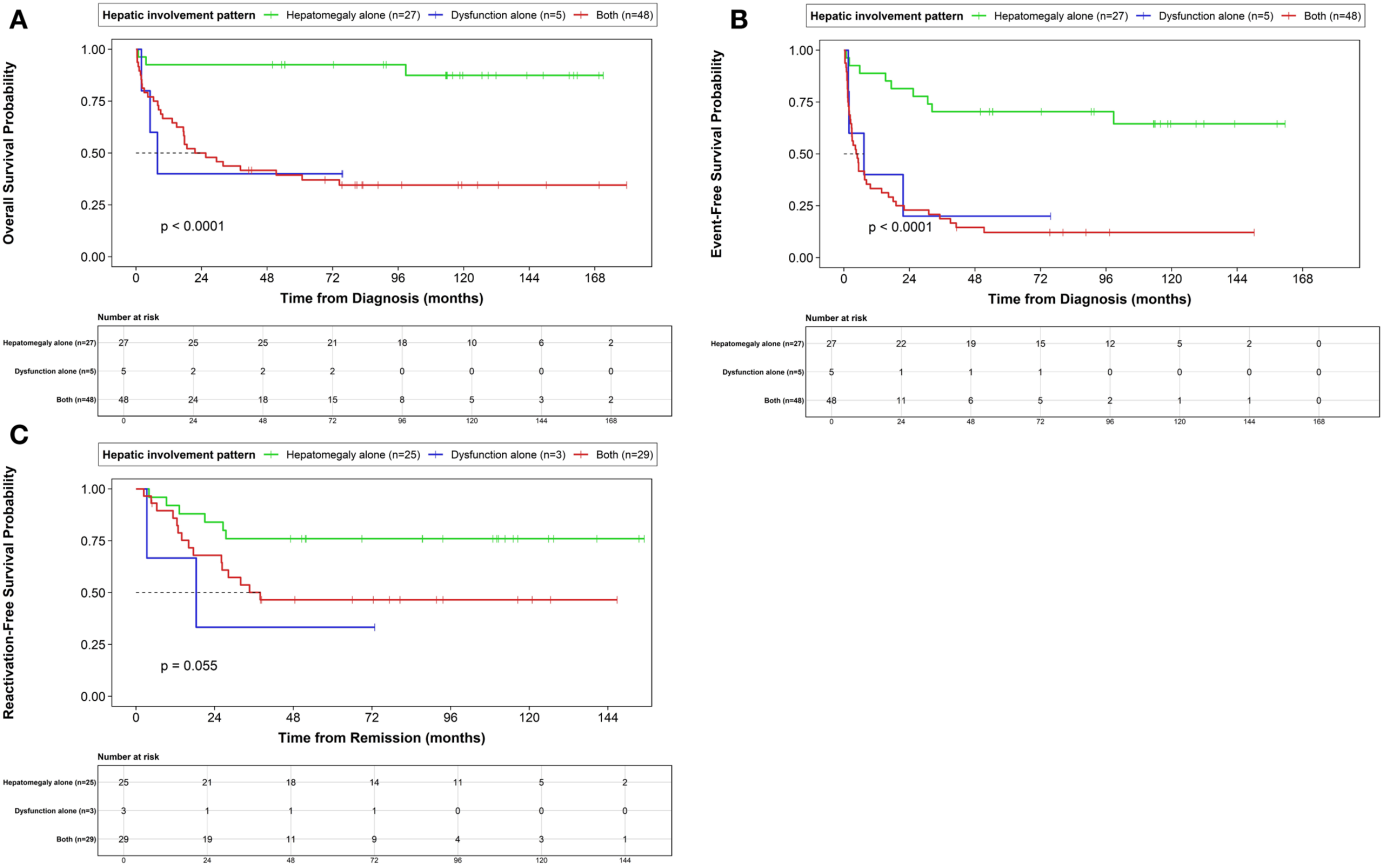
Hepatic dysfunction drives inferior OS **(A)**, EFS **(B)**, and RFS **(C)** independent of hepatomegaly.

Further stratification by hepatic involvement pattern confirmed favorable outcomes in patients with hepatomegaly and preserved liver function, while hepatic dysfunction was associated with uniformly poor survival independent of liver size (**Figure 3**).

### 3.6 Risk factors influencing survival outcomes in patients with hepatic LCH

On univariate analysis, poorer EFS was associated with age<2 years at diagnosis, hepatic dysfunction, advanced hepatic fibrosis on US, involvement of the spleen and hematopoietic system, and lack of response at week 6. In multivariable analysis, only week 6 non-response remained an independent predictor of inferior EFS (HR 9.26; 95% CI, 4.12–20.84; p< 0.001) (**Table 3****).** Furthermore, week 6 non-response was the sole independent predictor of OS (HR 3.17; 95% CI, 1.37–7.31; p = 0.007) (**Supplementary Table 1****).** No significant predictors of RFS were identified, although the analysis was limited by a small number of events (**Supplementary Table 2****)**.

**Table 3:** Risk factors associated with event-free survival in patients with hepatic LCH.

	Univariate			Multivariate		
Risk Variables	HR (95% CI)	P-value	q-value ^1^	HR (95% CI)	P-value	q-value ^1^
Age at diagnosis (Years)						
≥2 (Ref) <2	2.14 (1.18- 3.89)	0.013	0.013	1.69 (0.88 -3.23)	0.116	0.231
Liver Dysfunction						
No (Ref) Yes	5.52 (2.64-11.54)	<0.001	<0.001	2.36 (0.96- 5.79)	0.061	0.183
Associated Hematopoietic System						
No (Ref) Yes	3.63 (2.03- 6.49)	<0.001	<0.001	1.16 (0.51 - 2.64)	0.719	0.863
Associated Splenomegaly						
No (Ref) Yes	2.64 (1.33- 5.27)	0.006	0.006	1.05 (0.48 - 2.31)	0.901	0.901
US late fibrotic stage						
No (Ref) Yes	2.22(1.18 - 4.15)	0.013	0.013	0.72 (0.31- 1.71)	0.463	0.694
Pulmonary involvement						
No (Ref) Yes	0.89 (0.42 -1.88)	0.8	0.8	Not included	-	-
Early Response at W6						
Response (Ref) Non-response	12.6 (6.62-24.03)	<0.001	<0.001	9.26 (4.12 – 20.8)	<0.001	<0.001

1 False discovery rate (FDR) correction for multiple testing.

CI, Confidence Interval; HR, Hazard Ratio; US, Ultrasonography.

#### 3.6.1 Liver biochemical abnormalities associated with poor outcomes

Hypoalbuminemia and elevated direct bilirubin, transaminases, GGT, and ALP were linked to worse EFS in univariable analyses. Due to collinearity between GGT and ALP, they were combined into a single cholestatic enzyme variable for multivariable modeling. After adjustment, hypoalbuminemia remained an independent predictor of inferior EFS (HR 4.40; 95% CI, 1.52–12.7; p = 0.003), while elevated direct bilirubin showed a nonsignificant trend toward poorer outcomes (HR 2.52; 95% CI, 0.94–6.75; p = 0.056) (**Table 4****).**

**Table 4:** Liver biochemical predictors of event-free survival.

	Univariate			Multivariate		
	HR (95% CI)	P-value	q-value ^1^	HR (95% CI)	P-value	q-value ^1^
Hypoalbuminemia						
No (Ref) Yes	4.44 (2.09 -9.41)	<0.001	<0.001	4.4 (1.52 -12.7)	0.003	0.011
Elevated DBIL						
No (Ref) Yes	3.85 (2.17 - 6.83)	<0.001	<0.001	2.52 (0.94- 6.75)	0.056	0.11
Elevated serum transaminases						
No (Ref) Yes	3.31 (1.74 - 6.32)	<0.001	<0.001	1.87 (0.73- 4.78)	0.2	0.2
Elevated GGT						
No (Ref) Yes	3.02 (1.44 - 6.36)	0.004	0.004	Not included	-	-
Elevated ALP						
No (Ref) Yes	3.77 (2.07- 6.84)	<0.001	<0.001	Not included	-	-
Elevated cholestatic enzymes (GGT and/or ALP)						
No (Ref) Yes	Not included	-	-	0.71 (0.22-2.35)	0.6	0.6

1 False discovery rate (FDR) correction for multiple testing; CI= Confidence Interval, HR = Hazard Ratio,.

GGT, gamma-glutamyl transferase; ALP, alkaline phosphatase; DBIL, Direct bilirubin.

GGT and ALP were combined into a composite cholestatic enzyme variable in the multivariable model because of collinearity.

### 3.7 Extent of hepatomegaly and survival in patients with preserved liver function

**Figure 4:**
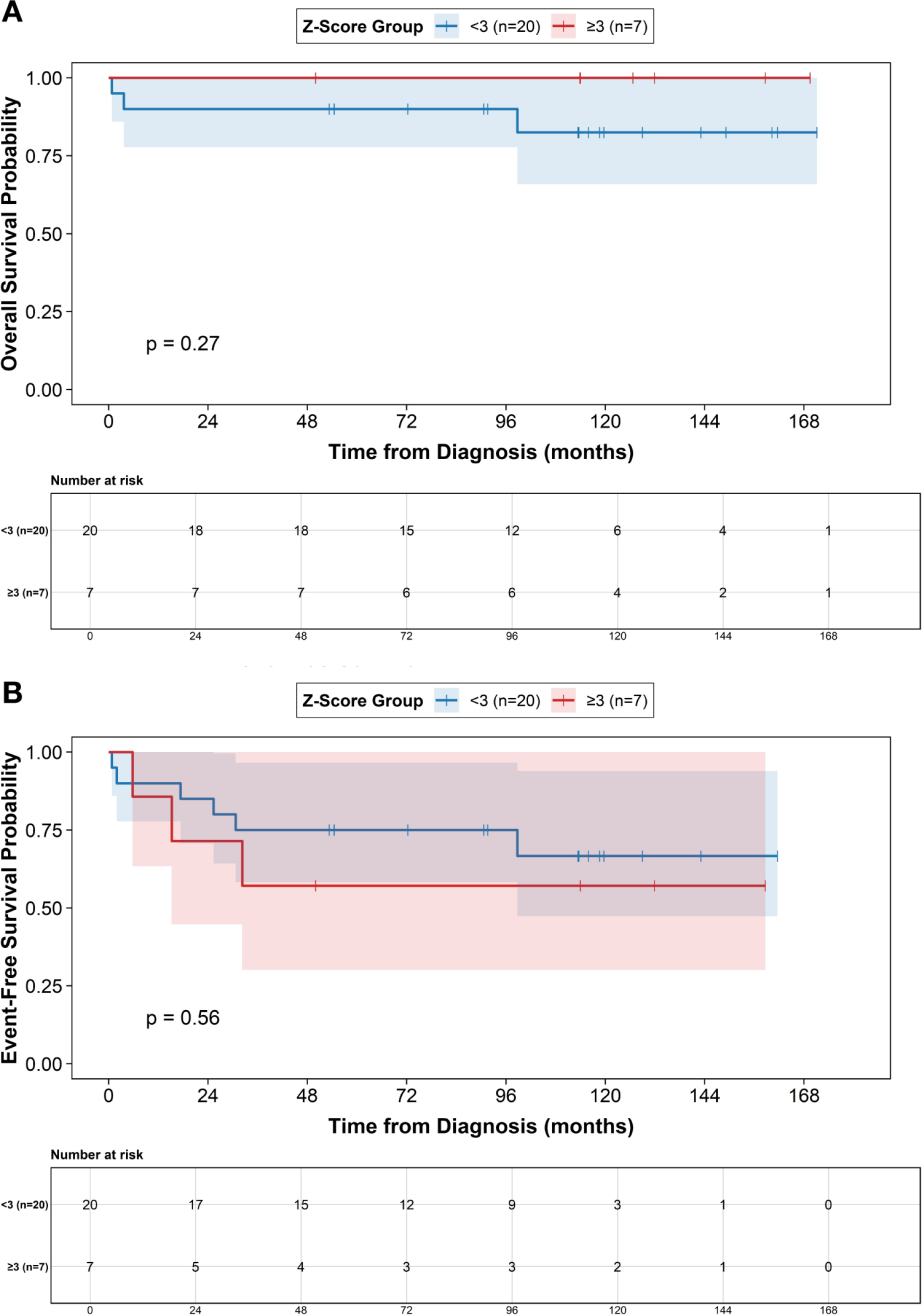
Survival outcomes stratified by extent of hepatomegaly in patients with preserved liver function [**(A)** OS; **(B)** EFS].

Among patients with hepatomegaly and preserved liver function, outcomes were further examined according to the degree of liver enlargement. No significant differences in the 5-year OS or EFS were observed between patients with massive hepatomegaly (n = 7) and those with mild–moderate hepatomegaly (n = 20) (log-rank p = 0.27, and p = 0.56, respectively; **Figure 4**).

## 4 Discussion

In the present study, liver involvement was identified in 17.6% of pediatric LCH cases and accounted for 43% of multisystem disease. It was significantly associated with younger age at diagnosis and with concurrent involvement of multiple organs, including the skin, lymph nodes, spleen, and hematopoietic system. These findings suggest that liver involvement characterizes an early-onset MS-LCH phenotype rather than isolated SS disease, consistent with previous reports (6, 12, 25).

Liver involvement in pediatric LCH most commonly manifests as hepatomegaly with biochemical abnormalities. Hepatic enlargement may result from direct LCH infiltration or immune-mediated mechanisms, and disease progression can persist despite apparent regression of active lesions. Radiologic findings vary by histopathologic stage, from proliferative and granulomatous to xanthomatous and fibrotic phases, with predominant periportal involvement that may be missed on US but is more reliably detected by CT or magnetic resonance imaging (MRI), even in the absence of overt liver dysfunction. Prior studies have shown that integrating US with cross-sectional imaging enhances diagnostic yield, highlighting the complementary role of CT and MRI when initial US findings are negative or indeterminate (15, 25).

In our cohort, diagnostic evaluation relied primarily on US, reflecting limited access to cross-sectional imaging, particularly during the early study period. Furthermore, the absence of magnetic resonance cholangiopancreatography (MRCP), the reference standard for diagnosing sclerosing cholangitis (26), necessitated diagnosis based on characteristic fibrotic US features combined with cholestatic biochemical abnormalities, following previously established diagnostic criteria (12).

SC was identified in 18.75% of cases, compared with 33.8% reported by Ge et al. (12) and 30% reported by Braier et al. (27). This variability likely reflects heterogeneity in diagnostic criteria, differences in imaging sensitivity, and variation in the severity and extent of hepatic involvement across study cohorts.

Liver involvement in LCH was associated with significantly inferior outcomes, with markedly reduced 5-year OS and EFS, in line with previous reports, underscoring liver disease as a major adverse prognostic factor (12, 25). This unfavorable prognosis likely reflects a convergence of factors, including advanced and potentially irreversible hepatic injury at presentation, the development of SC, and the lack of durable options for refractory disease. Notably, outcomes remained poor even in the LCH-IV era following the introduction of 2-CdA BR, particularly in those who developed disease reactivation, suggesting that the timing of treatment escalation is critical. These findings indicate that while 2-CdA BR is effective in high-risk LCH overall, its benefit appears substantially diminished once progressive hepatic disease or reactivation has occurred (14). This pattern supports the concept that hepatic LCH represents a distinct, biologically aggressive, and treatment-resistant phenotype, in which disease control may be insufficient to prevent organ failure–related mortality.

Although molecular profiling was not available in this cohort, emerging data indicate enrichment of BRAF V600E and other MAPK pathway–activating alterations in hepatic LCH, which may contribute to chemoresistance. These findings support earlier risk stratification, prompt therapeutic escalation before irreversible damage, and consideration of targeted or liver-directed approaches in this high-risk subgroup; however, uncertainties regarding reactivation following treatment discontinuation and substantial financial burden continue to limit their widespread application, particularly in low- and middle-income settings (28).

Furthermore, stratified analyses demonstrated that hepatic dysfunction conferred adverse outcomes irrespective of hepatomegaly, establishing functional hepatic impairment as the principal prognostic determinant. This association with poor survival likely reflects advanced hepatic involvement with extensive LCH infiltration and progressive hepatobiliary injury, reflecting hepatic damage that may limit reversibility despite therapy. Notably, the development of SC did not significantly influence survival among patients with established hepatic dysfunction. This finding may reflect the uniformly limited availability of effective salvage therapies for advanced hepatic dysfunction in our cohort, potentially leading to comparably poor outcomes regardless of SC status and obscuring survival differences between subgroups. In addition, the relatively small number of SC cases and the reliance on US-based criteria without confirmatory cross-sectional imaging may have limited statistical power and introduced potential classification bias, therefore diminishing the ability to detect subtle prognostic differences.

In multivariable models, liver dysfunction emerged as the only significant predictor of poor response to initial therapy, whereas the choice of induction regimen (LCH-III vs. LCH-IV) had no measurable effect. The lack of a protocol-specific benefit likely reflects the limited impact of ID-MTX on induction efficacy, consistent with previously published data showing that the randomized addition of MTX to PRED and VBL in patients with LCH-RO+ did not improve response to initial therapy or survival (22).

In this cohort, early treatment response at week 6 was the strongest determinant of outcome. In multivariable analysis, baseline risk factors including age at diagnosis, hepatic dysfunction, ultrasonographic evidence of advanced hepatic fibrosis, and concomitant hematopoietic and spleen involvement did not retain independent prognostic significance, whereas non-response at week 6 remained an independent predictor of both EFS and OS. These findings are in line with results from Histiocyte Society studies, including the LCH-III trial reported by Gadner et al. (22) which identified early treatment response at week 6 as the most robust predictor of outcome in multisystem LCH. In these studies, the apparent prognostic impact of baseline characteristics, especially younger age, diminished after accounting for treatment response and risk organ involvement. Our results support this paradigm, as age<2 years was not independently associated with outcome once early response at week 6 was considered, suggesting that younger age reflects a higher burden of aggressive disease rather than acting as an independent prognostic factor. Similarly, although involvement of additional risk organs, such as the spleen and hematopoietic system, was associated with adverse outcomes in univariable analyses, these factors did not remain significant in adjusted models. This may reflect overlap among markers of disease burden, as well as limited statistical power related to a low number of events, potentially reducing the ability to detect independent associations. Additionally, the prognostic impact of risk-organ involvement may be partially mediated through early treatment response, leading to attenuation of its effect after adjustment (22, 29).

Among the laboratory parameters, hypoalbuminemia remained independently associated with inferior EFS, underscoring the prognostic relevance of impaired hepatic synthetic function.

In hepatic LCH, presentation in the context of preserved liver function represents a relatively uncommon phenotype compared with cases complicated by hepatic dysfunction. In our cohort, this subgroup was associated with favorable long-term outcomes, with 5-year OS, EFS, and RFS rates of 92.6% ± 5.0%, 70.4% ± 8.8%, and 76.0% ± 8.5%, respectively. Using age-standardized assessment of liver size, stratification by the degree of hepatic enlargement revealed no significant survival differences between patients with massive hepatomegaly (Z-score ≥ 3 SD) and those with mild-to-moderate enlargement (Z-score > +2 to< +3 SD). These findings indicate that, in the absence of hepatic functional impairment, the extent of hepatomegaly alone does not confer additional prognostic risk. Collectively, these observations raise the possibility that hepatic dysfunction may be more relevant than hepatomegaly in defining high-risk liver involvement. However, given the relatively small size of the subgroup with preserved liver function, these findings should be interpreted with caution and considered hypothesis-generating.

This study represents one of the earliest systematic evaluations of survival across distinct hepatic phenotypes in LCH, a setting in which liver involvement has historically been analyzed as a single entity and supports the prioritization of hepatic dysfunction over hepatomegaly when defining high-risk liver involvement, pending validation in larger, multicenter, prospective cohorts.

This retrospective study is limited by incomplete availability of cross-sectional imaging and MRCP, which may have led to under-recognition of early periportal or biliary involvement; liver size Z-scores were derived from European reference standards, as population-specific normative data were not applicable; and lack of molecular profiling limited evaluation of genomic drivers of adverse outcomes. Finally, given the rarity of pediatric LCH, the small sample size and limited number of events reduced statistical power, particularly in subgroup analyses. Covariates were restricted according to the EPV rule, particularly in the RFS analysis, which should be considered exploratory and may have led to exclusion of relevant variables. Wide confidence intervals indicate imprecision due to sparse data. These findings should be interpreted with caution and require validation in larger cohorts.

In conclusion, hepatic dysfunction, rather than hepatomegaly with preserved liver function, was associated with adverse outcomes in LCH. Among evaluated risk factors, non-response at week 6 was independently associated with inferior event-free survival and overall survival. In contrast, patients with hepatomegaly but preserved hepatic function demonstrated favorable outcomes regardless of liver size; however, this observation should be interpreted cautiously given the small subgroup size. These findings may inform future refinement of risk stratification models and suggest a shift toward function-based definitions of high-risk hepatic involvement. Larger, multicenter prospective studies are warranted to validate these observations.

## Data Availability

The original contributions presented in the study are included in the article/**Supplementary Material**. Further inquiries can be directed to the corresponding authors.
