# Supplementary material for: Structural and functional hepatic factors as prognostic indicators in children with Langerhans cell histiocytosis
Source: Front Oncol. 2026 May 29;16:1813004. doi: 10.3389/fonc.2026.1813004 (PMC13259843; doi:10.3389/fonc.2026.1813004)
Supplement: Supplementary file 1 [file DataSheet1.docx]

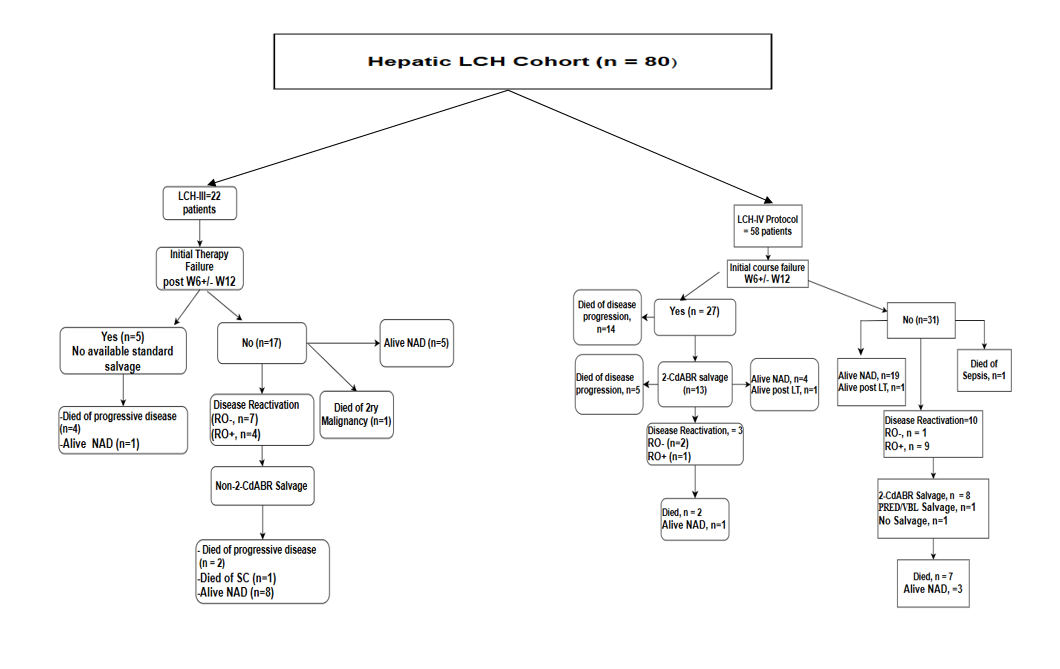


**Supplementary Figure S2: Treatment Outcome Among Patients with Hepatic LCH**

**LT: Liver Transplantation; NAD: Non- active disease; RO-: Non-Risk organ; RO+: Risk Organ; SC: Sclerosing cholangitis.**
