# Supplementary material for: Structural and functional hepatic factors as prognostic indicators in children with Langerhans cell histiocytosis
Source: Front Oncol. 2026 May 29;16:1813004. doi: 10.3389/fonc.2026.1813004 (PMC13259843; doi:10.3389/fonc.2026.1813004)
Supplement: Supplementary file 2 [file DataSheet2.docx]

| **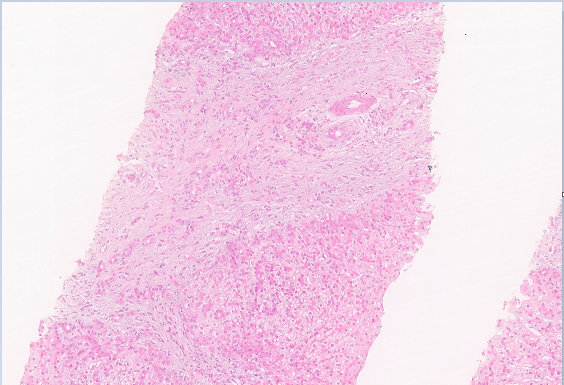**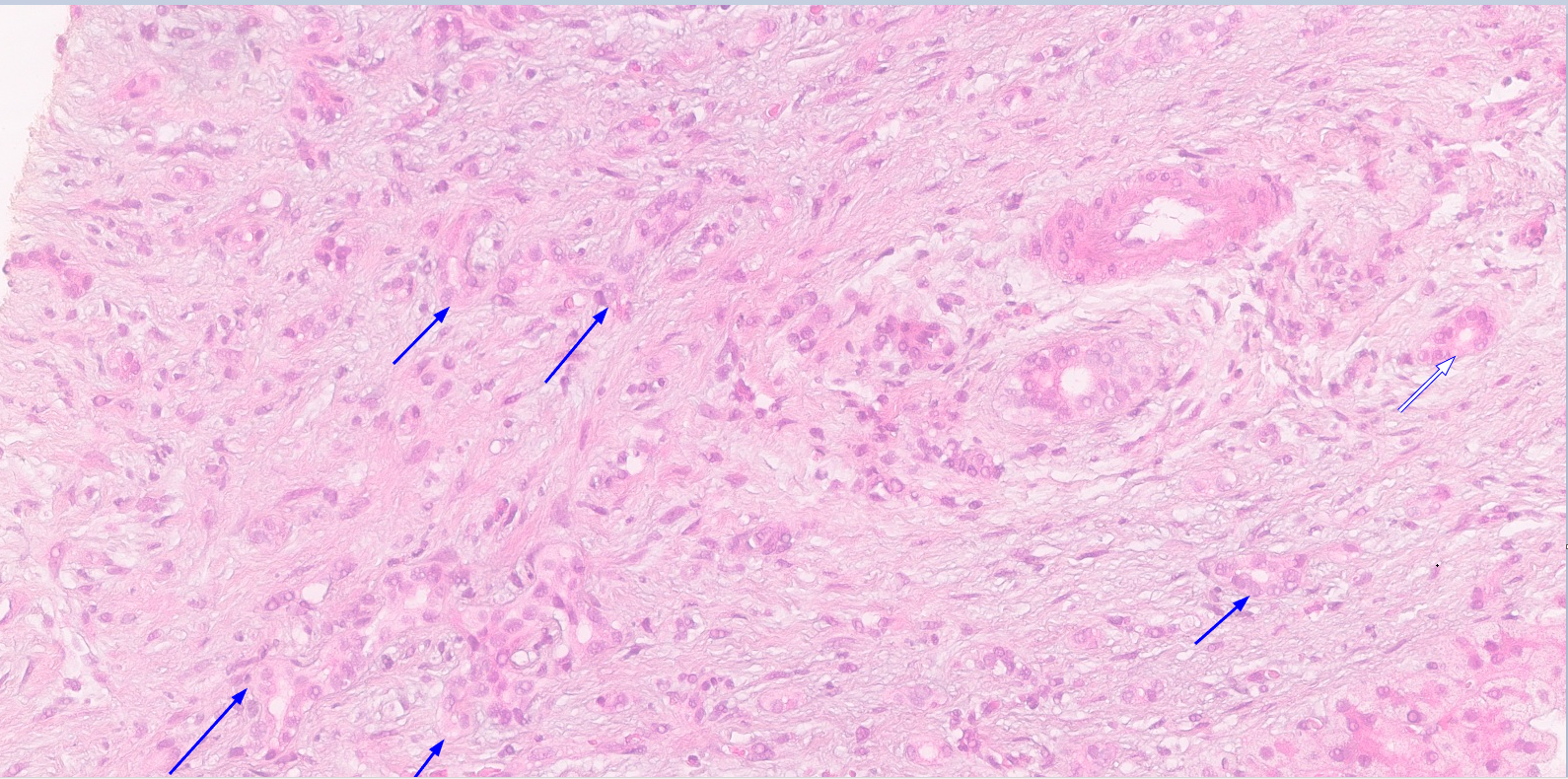  **(A) (B)**    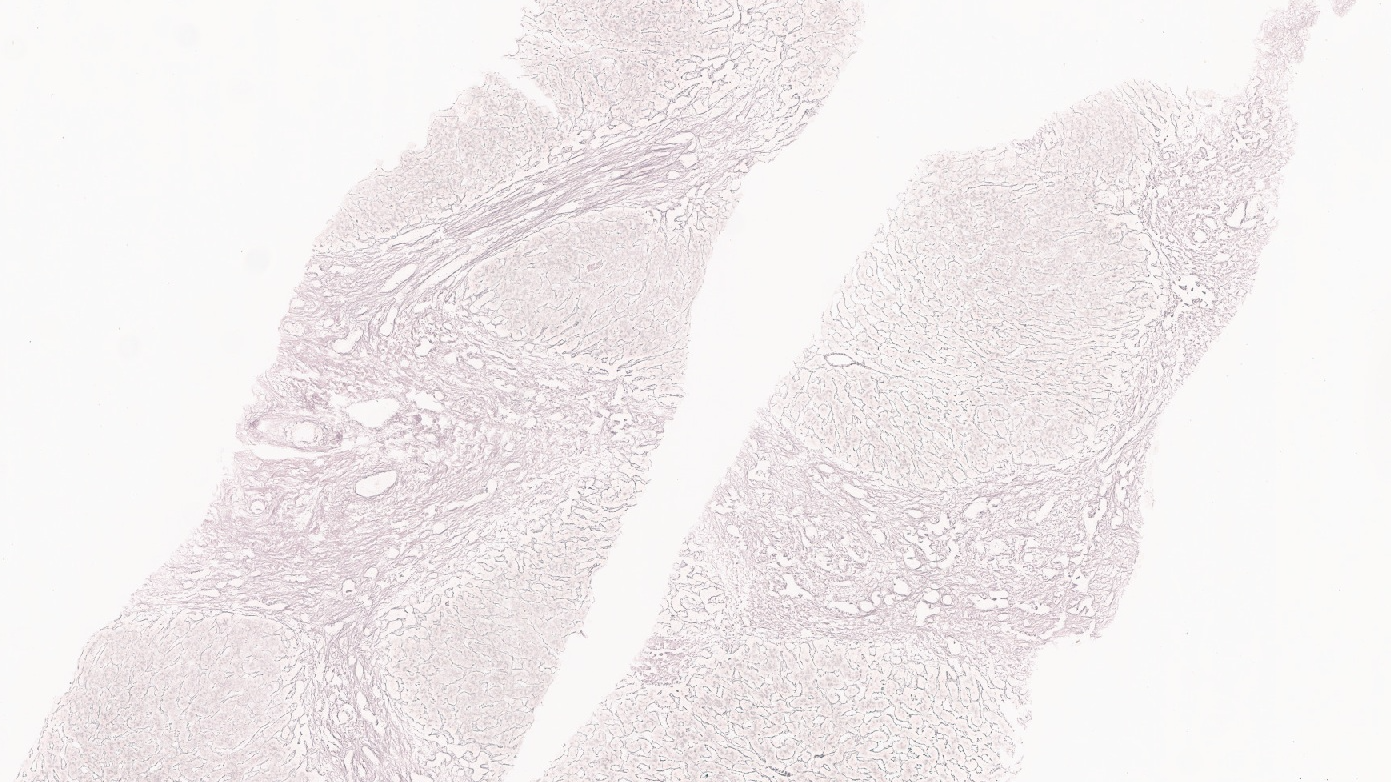  **(C)** |
| --- |

**Supplementary Figure S1:** Histopathologic features of liver biopsy in a case with hepatic LCH: **(A):** H&E x100: Highlights portal tract expansion by mild inflammatory cell infiltrate, fibrosis and bile duct proliferation, **(B):** H&E x200: Higher power with arrows highlighting some of the proliferating bile ducts. **(C):** Reticulin stains: Highlights broad bands of bridging fibrosis with nodule formation (stage 4 fibrosis).

**Supplementary Table S1: Risk factors associated with overall survival in patients with hepatic LCH**

|  | **Univariate** | | | **Multivariate** | | |
| --- | --- | --- | --- | --- | --- | --- |
| **Risk Variables** | **HR**  **(95% CI)** | **P-value** | **q-value ^1^** | **HR**  **(95% CI)** | **P-value** | **q-value ^1^** |
| **Age at diagnosis (Years)** |  |  |  |  |  |  |
| **≥2 (Ref)**  **<2** | **1.87(0.90- 3.87)** | **0.091** | **0.091** | **-** |  |  |
| **Liver Dysfunction** |  |  |  |  |  |  |
| No (Ref)  Yes | 8.79 (2.68-28.8) | <0.001 | <0.001 | 3.84 (1.01- 14.61) | 0.048 | **0.097** |
| **Associated Hematopoietic System** |  |  |  |  |  |  |
| No (Ref)  Yes | 2.82 (1.44- 5.50) | 0.002 | 0.002 | 1.35 (0.59-3.08) | 0.474 | 0.474 |
| **Associated Splenomegaly** |  |  |  |  |  |  |
| No (Ref)  Yes | 3.24 (1.26 – 8.33) | 0.015 | 0.015 | ^*^Not Included |  |  |
| **US late fibrotic stage** |  |  |  |  |  |  |
| No (Ref)  Yes | 2.59 (1.27 – 5.29) | 0.009 | 0.009 | 1.42 (0.60-3.36) | 0.423 | 0.474 |
| **Pulmonary involvement** |  |  |  |  |  |  |
| No (Ref)  Yes | 0.89 (0.42 -1.88) | 0.8 | 0.8 | - |  |  |
| Early Response at W6 |  |  |  |  |  |  |
| Response (Ref)  Non-response | 6.18 (2.96-12.88) | <0.001 | <0.001 | 3.17 (1.37-7.31) | 0.007 | 0.028 |

^1^ False discovery rate (FDR) correction for multiple testing

CI= confidence interval, HR = hazard ratio, US= ultrasonography

^*^ Not included: Variables were selected based on model fit, with covariate number limited by the 10 events-per-variable rule to avoid overfitting.

**Supplementary Table S2: Risk factors associated with RFS in patients with hepatic LCH**

|  | **Univariate** | | | **Multivariate** | | |
| --- | --- | --- | --- | --- | --- | --- |
| **Risk Variables** | **HR**  **(95% CI)** | **P-value** | **q-value ^1^** | **HR**  **(95% CI)** | **P-value** | **q-value ^1^** |
| **Age at diagnosis (Years)** |  |  |  |  |  |  |
| **≥2 (Ref)**  **<2** | 1.73 (0.71-4.22) | 0.226 | 0.226 | 1.42 (0.56 – 3.59) | 0.462 | 0.672 |
| **Liver Dysfunction** |  |  |  |  |  |  |
| No (Ref)  Yes | 2.78 (1.09-7.06) | 0.032 | 0.032 | 2.78 (1.00-7.72) | **0.049** | **0.148** |
| **Associated Hematopoietic System** |  |  |  |  |  |  |
| No (Ref)  Yes | 2.21 (0.87-5.62) | 0.097 | 0.097 | ^*^Not Included |  |  |
| **Associated Splenomegaly** |  |  |  |  |  |  |
| No (Ref)  Yes | 2.37 (0.88- 6.39) | 0.089 | 0.089 | ^*^Not Included |  |  |
| **US late fibrotic stage** |  |  |  |  |  |  |
| No (Ref)  Yes | 1.22 (0.36 - 4.11) | 0.749 | 0.749 | ^*^Not Included |  |  |
| **Pulmonary involvement** |  |  |  |  |  |  |
| No (Ref)  Yes | 0.8 (0.24 -2.70) | 0.7 | 0.7 | ^*^Not Included |  |  |
| Early Response at W6 |  |  |  |  |  |  |
| Response (Ref)  Non-response | 1.48 (0.61 -3.61) | 0.385 | 0.385 | 0.81(0.30- 2.16) | 0.672 | 0.672 |

^1^ False discovery rate (FDR) correction for multiple testing

CI= confidence interval, HR = hazard ratio, US= ultrasonography

^*^ Not included: Variables were selected based on model fit, with covariate number limited by the 10 events-per-variable rule to avoid overfitting.

| 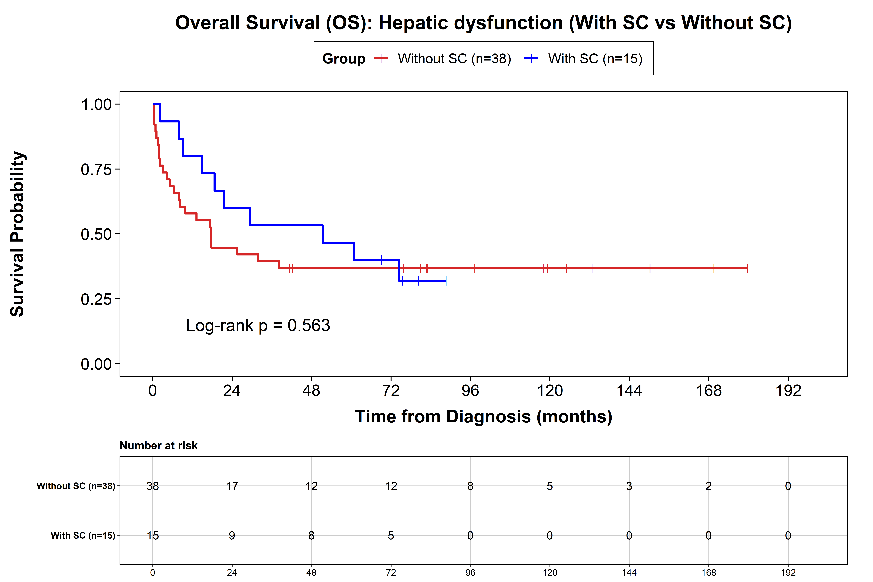 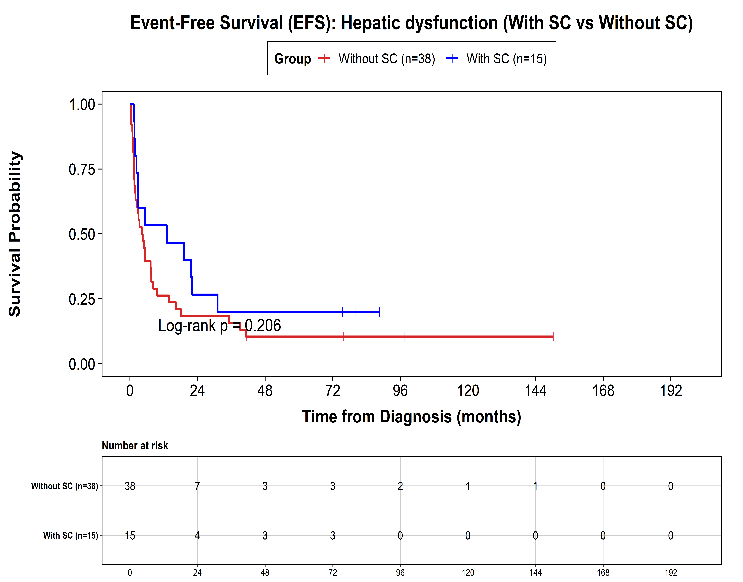 |
| --- |

**Supplementary Figure S3**: Overall and Event-Free Survival According to SC Status in Patients with Liver Dysfunction.
